# Brain structures associated with executive functions during everyday events in a non-clinical sample

**DOI:** 10.1007/s00429-012-0444-z

**Published:** 2012-08-01

**Authors:** Hikaru Takeuchi, Yasuyuki Taki, Yuko Sassa, Hiroshi Hashizume, Atsushi Sekiguchi, Ai Fukushima, Ryuta Kawashima

**Affiliations:** 1 Smart Ageing International Research Center, Institute of Development, Aging and Cancer, Tohoku University, 4-1, Seiryo-cho, Aoba-ku, Sendai, 980-8575 Japan; 2 Division of Developmental Cognitive Neuroscience, Institute of Development, Aging and Cancer, Tohoku University, Sendai, Japan; 3 Department of Functional Brain Imaging, Institute of Development, Aging and Cancer, Tohoku University, Sendai, Japan

**Keywords:** Executive functions during everyday events, Voxel-based morphometry, Regional gray matter volume, Regional white matter volume, Orbitofrontal cortex

## Abstract

Executive functions involve control processes such as goal-oriented planning, flexible strategy generation, sustaining set maintenance, self-monitoring, and inhibition. Executive functions during everyday events (EFEEs) are distinct from those measured under laboratory settings; the former can be severely impaired while the latter remain intact. Non-routine everyday problems due to executive dysfunctions affect individual functioning in everyday life and are of great clinical interest. Despite the importance of anatomical bases underlying better EFEEs, such bases have never been investigated among non-clinical samples. Using voxel-based morphometry to measure regional gray matter volume (rGMV) and regional white matter volume (rWMV) and diffusion tensor imaging to determine fractional anisotropy values, we identified the anatomical correlates of better EFEEs using the Dysexecutive Questionnaire in 303 normal young subjects (168 men and 135 women). Better EFEEs were associated with a smaller rGMV in the orbitofrontal cortex (OFC) spread across Brodmann areas (BA) 25, 11, and 12 and larger rWMV in the WM area of OFC adjacent to BA 11. Furthermore, individual EFEEs were positively associated with rWMV in the temporal areas, primarily the inferior longitudinal fasciculus and inferior fronto-occipital fasciculus, the latter of which connects OFC and posterior regions. Thus, our findings suggest that brain structures involving OFC, together with other regions, contribute to the maintenance of effective EFEEs among non-clinical subjects.

## Introduction

While executive functions involve control processes such as goal-oriented planning, flexible strategy generation, sustaining set maintenance, self-monitoring, and inhibition, executive dysfunction refers to the degradation of these cognitive abilities (Cooney et al. 2004; Luria and Haigh 1973; Shallice and Burgess 1991; Welsh and Pennington 1988). Anatomically, executive functions have been associated with functions of the prefrontal cortex (PFC) (Mesulam 2002), and there is now a consensus that damage to the frontal lobe causes impairment of executive functions (executive dysfunctions) and a disastrous inability to cope in many real-world situations (Chan and Manly 2002). Nevertheless, executive dysfunctions during everyday events are widely observed among non-clinical samples comprising subjects who have no history of psychiatric or neurological diseases and who do not have substance abuse disorders (Chan 2001a). In addition, increasing evidence suggests that conditions associated with executive dysfunction, such as autistic spectrum disorders and attention-deficit hyperactivity disorder, are on a continuum with normality (Baron-Cohen et al. 2003; Levy et al. 1997). Executive functions during everyday events (EFEEs) are distinct from those measured under laboratory settings (Damasio 1994; Eslinger and Damasio 1985; Shallice and Burgess 1991). The former can be selectively and severely impaired causing serious behavioral problems in real life whereas the latter, which is measured by working memory and rule-switching tasks, remains intact (Damasio 1994; Eslinger and Damasio 1985; Shallice and Burgess 1991). This discrepancy suggests that the two have distinctive neural correlates. Judging from case studies of selective impairments of EFEEs (Damasio 1994; Eslinger and Damasio 1985), cognitive functions that are specifically involved in EFEEs may include making correct decisions in situations associated with complex information input and inhibiting socially inappropriate behaviors in socially complex situations that sometimes involve implicit information. Recent advances in neuroimaging and non-neuroimaging studies have introduced the dichotomy of ‘cool’ versus ‘hot’ executive functions (Carlson 2005; Haber 2003; Zelazo and Müller 2002). While cool executive functions include working memory, planning, and problem solving, hot executive functions involve affective decision making or decision making about events that have emotionally significant consequences (Hongwanishkul et al. 2005; Kerr and Zelazo 2004; Poletti 2010). As such, hot executive functions may at least partly correspond to EFEEs. Furthermore, cold executive functions are associated with dorsolateral regions of PFC whereas hot executive functions are associated with the orbital regions of PFC (Zelazo and Müller 2002).

Non-routine everyday problems because of executive dysfunctions affect an individual’s daily functions and are of great clinical interest. Despite their importance, anatomical bases underlying better EFEEs among non-clinical samples have never been investigated. The purpose of this study was to investigate the association between brain structures and EFEEs among non-clinical subjects. Individual differences in executive functions play a role in building and maintaining a successful everyday life. Investigating the anatomical bases underlying EFEEs is important because it provides new insights into how a successful everyday life can be achieved and ideas for clinical applications to assess executive dysfunctions during everyday events.

In this study, we focused on three aspects of brain structures: regional gray matter volume (rGMV), regional white matter volume (rWMV), and fractional anisotropy (FA) determined by diffusion tensor imaging (DTI). Potential correlates of rGMV may include the number and size of neurons and glias, the level of synaptic bulk, and the number of neurites (Draganski et al. 2004; May and Gaser 2006). Furthermore, rGMV/rWMV is known to be associated with various cognitive abilities, and by investigating these, we can identify the associated brain regions (e.g., Brickman et al. 2006; Hänggi et al. 2010; Haier et al. 2004; Johnson et al. 2007; Takeuchi et al. 2010a). As summarized in our previous study (Takeuchi et al. 2011d), in DTI, the FA in each voxel is used as a measure of the degree of diffusion anisotropy, with FA reflecting the angle (degree of directionality) of cellular structures within the fiber tracts, and therefore, reflecting the structural integrity (Chua et al. 2008). FA has previously been used as a measure of structural integrity (but crossing fibers can appear as a voxel of low anisotropy; Tuch et al. 1999). Consistent with this notion, a pathological postmortem study showed (Schmierer et al. 2007) that FA value was strongly correlated with the amount of myelin. Cognitive processing speed, which has been assumed to be associated with the structures of the white matter pathways (Turken et al. 2008), is positively correlated with FA in various regions (Tuch et al. 2005; Turken et al. 2008). FA ranges from 0, representing isotropic diffusion, to 1, where diffusion takes place only in one direction. FA and rWMV were moderately to weakly related, but they were highly sensitive to the different characteristics of white matter (Fjell et al. 2008). The associations between the two seem particularly weak in deep white matter areas (Hugenschmidt et al. 2008). Distributions of the associations between FA and group/individual differences and distributions of the associations between rWMV and group/individual differences differ sometimes (Hugenschmidt et al. 2008; Jäncke et al. 2009). Thus, by utilizing both methods, we are able to investigate different neural mechanisms. Thus, these anatomical imaging methods provide unique and distinct information regarding the origin of EFEEs. We used the Dysexecutive Questionnaire (DEX; Burgess et al. 1998) to assess individual executive (dys)functions during everyday events. Our hypothesis was that brain structures involving the orbitofrontal cortex (OFC), namely rGMV, rWMV, and FA of OFC, and rWMV and FA of the anterior part of the corpus callosum, which connects the bilateral OFC and other white matter regions adjacent to OFC (Huang et al. 2005), would be associated with individual EFEEs as measured by DEX among the non-clinical subjects.

## Methods

### Subjects

A total of 303 healthy, right-handed individuals (168 men and 135 women) were included in this study, which is a part of our ongoing project to investigate the association among brain imaging, cognitive function, and aging (Takeuchi et al. 2010a, b, c, 2011a; Taki et al. 2010, 2011). Most of the subjects who participated in this study had also participated in our intervention studies (Takeuchi et al. 2011b, c). In these intervention studies, participants underwent cognitive training and the effects of the training were investigated (psychological and imaging data recorded before intervention were used in the present study). They completed psychological tests and questionnaires, which did not include measures of extraversion and impulsivity, and underwent MRI scans that are not described in this study but were performed together with other scans, described in this study. The mean age of the subjects was 21.2 years [standard deviation (SD) 1.74]. All subjects were college, university, postgraduate students or within 1-year after graduation of those. Most of the subjects were associated with Tohoku University. They were recruited using advertisements on bulletin boards at Tohoku University or via email introducing the study to subjects who had participated in our previous experiments. One subject who had recently been subjected to psychological measures used in this study was excluded from further analysis. Thus, the effects of repeated exposure to psychological measures used in this study can be ignored. None of the subjects had visual difficulties, hearing difficulties, speech impairments, motor impairments, substance abuse disorders, or a history of neurological or psychiatric illness. These characteristics of the subjects were the same as those of our previous study (Chan 2001a). Handedness was evaluated using the Edinburgh Handedness Inventory (Oldfield 1971). In accordance with the Declaration of Helsinki (1991), written informed consent was obtained from each subject. This study was approved by the Ethics Committee of Tohoku University.

### Assessment of EFEEs (behavioral dysexecutive symptoms)

DEX is a part of the Behavioral Assessment of the Dysexecutive Syndrome (BADS) (Wilson et al. 1996), which is considered a sensitive and ecologically valid measure of dysexecutive symptoms among patients with different types of neurological disorders (Burgess et al. 1998; Wilson et al. 1998) as well as among non-clinical subjects (Chan 2001a). DEX can quantify EFEEs but not executive functions under laboratory settings. Although DEX was developed for clinical purposes, it can be used to identify neural correlates of EFEEs among non-clinical samples as well as the number of other psychological measures or tasks that were developed for clinical purposes but can be used for young healthy adults (e.g., see Baron-Cohen et al. 1985 for the Sally–Anne task; Sommer et al. 2007). We administered the Japanese version of DEX, which is part of the Japanese version of BADS (Kashima 2003).

Participants were asked to complete the self-rated version of DEX. DEX is a 20-item questionnaire designed to assess everyday signs of dysexecutive symptoms. The instructions provided to the participants were to read the 20 statements describing common problems associated with everyday events and rate them according to their personal experience. Each item was scored on a five-point scale according to frequency ranging from “never” (zero points) to “very often” (four points). All items of DEX are provided in Table 1. An overall impairment score was calculated by adding the 20 individual item scores (the maximum score was 80). Thus, lower DEX scores indicated higher EFEEs. To the best of our knowledge, DEX does not have a threshold for clinical diagnosis. The reliability of this measure in the present study population was as follows. The internal consistency of this measure was 0.896 (Cronbach’s coefficient of α). Furthermore, the correlation coefficient between DEX items with odd and even numbers obtained using the split-half method was *r* = 0.920.

**Table 1:** The 20 DEX statements

	Item	Factor loadings
1	I have problems understanding what other people mean unless they keep things simple and straightforward	0.479
2	I act without thinking, doing the first thing that comes to mind	0.434
3	I sometimes talk about events or details that never actually happened, but I believe they did happen	0.515
4	I have difficulty thinking ahead or planning for the future	0.596
5	I sometimes get over-excited about things and can be a bit over the top at times	0.619
6	I get events mixed up with each other and get confused about the correct order of events	0.551
7	I have difficulty realizing the extent of my problems and am unrealistic about the future	0.611
8	I seem lethargic and unenthusiastic about things	0.499
9	I do or say embarrassing things when in company of others	0.536
10	I really want to do something one minute but could not care less about it the next	0.555
11	I have difficulty showing emotion	0.494
12	I lose my temper at the slightest thing	0.393
13	I seem unconcerned about how I should behave in certain situations	0.570
14	I find it hard to stop repeating, saying, or doing things once started	0.543
15	I tend to be very restless and I cannot sit still for any length of time	0.577
16	I find it difficult to stop doing something, even if I know I should not.	0.678
17	I will say one thing but will do something different	0.710
18	I find it difficult to keep my mind on something and am easily distracted	0.642
19	I have trouble making decisions or deciding what I want to do	0.535
20	I am unaware of, or unconcerned about, how others feel about my behaviors	0.498

### Factor analysis of DEX

To determine whether analysis of total DEX scores was sufficient or whether multiple factors associated with DEX, and additional analysis using those multiple factors were required, we employed factor analysis.

Promax-rotated factor analysis (unweighted least squares method) of scores for each question in DEX was performed. Based on the recommendation of Stevens (1996), the Scree test was used to determine the number of factors. As a result, one-factor solution was selected. Eigenvalues of the first five factors were 6.87, 1.45, 1.11, 1.06, and 0.98. The eigenvalue of the first factor was clearly different from that of the other factors, supporting the use of a unitary construct. This factor solution is not consistent with that of a previous study conducted among non-clinical subjects (five-factor solution) (Chan 2001b). However, the difference may be largely due to the methods employed to perform factor analysis. In the previous study, eigenvalue of the first factor was also clearly different (in the previous study eigenvalues of the first five factors were 7.14, 1.75, 1.57, 1.12, and 1.01). However, the Kaiser–Guttman rule was applied to determine the number of factors in the previous study, and therefore, the five-factor solution was selected. Thus, the apparent saliency of the first factor was common between the present and the previous study.

EFEEs may be associated with a number of components and their corresponding neural bases; however, as far as DEX (at least among the non-clinical subjects) scores are concerned, the effect of the first factor is outstanding. Besides the fact that DEX was constructed to use total scores, and that total scores have been typically used in other studies involving DEX (Evans et al. 1997; Heffernan et al. 2004), the results of this factor analysis support the use of total scores. The factor loadings are shown in Table 1. Based on these factor loadings, we believed that we could include all items in the analyses. The recommended cutoff value of factor loadings to include certain items in the factor, recommended is sometimes >0.4 (Ishii 2005), and when this cutoff value is applied, item 12 in this study would be excluded. However, when the number of subjects is large, as in our study, a high cutoff value need not to be used and a cutoff value of approximately 0.35 is considered suitable (Ishii 2005).

### Assessment of general intelligence

To exclude the possibility that the association between brain structures and EFEEs is because of an association between brain structures and more general cognitive functions, we assessed general intelligence. Like any validated psychological measure, DEX scores correlate with other psychological measures including psychometric measures of intelligence in clinical samples (Chaytor and Schmitter-Edgecombe 2007; Wood and Liossi 2006). Raven’s Advanced Progressive Matrices (RAPM) is a psychometric measure of general intelligence, and it is often shown to be the measure most associated with general intelligence (Raven 1998). Thus, we used this test to assess general intelligence, which refers to the *g* factor (Spearman 1904) that contributes to success in diverse forms of cognitive tests regardless of whether they are verbal or non-verbal. This test was used here to adjust for the effect of individual psychometric measures of intelligence on brain structures. It was also performed because we have to exclude the possibility that any significant association between brain structures and EFEEs is because of an indirect association between brain structures and general intelligence (e.g., Colom et al. 2006; Haier et al. 2005; Jung and Haier 2007; Narr et al. 2007; Shaw et al. 2006). The test contains 36 non-verbal items requiring fluid reasoning ability. Each item consists of a 3 × 3 matrix with a missing piece to be completed by selecting the best of eight alternatives. The score from this test (number of correct answers given in 30 min) was used as a psychometric index of individual intelligence.

### Image acquisition

All MRI data acquisition were performed using a 3T Philips Achieva scanner. A magnetization-prepared rapid acquisition gradient echo sequence was used to collect high-resolution T1-weighted structural images (240 × 240 matrix, TR 6.5 ms, TE 3 ms, FOV 24 × 24 cm, number of slices 162, slice thickness 1.0 mm, flip angle 8°). Diffusion-weighted data were acquired using a spin-echo EPI sequence (TR 10,293 ms, TE 55 ms, big delta (Δ) 26.3 ms, little delta (δ) 12.2 ms, FOV 22.4 cm, 2 × 2 × 2 mm^3^ voxels, 60 slices, SENSE reduction factor 2, number of acquisitions 1). The diffusion weighting was isotropically distributed along 32 directions (*b* value = 1,000 s/mm^*2*^). In addition, three images with no diffusion weighting (*b* value = 0 s/mm^2^) (*b* = 0 images) and one *b* = 0 image were acquired from 175 and 127 subjects, respectively, using a spin-echo EPI sequence (TR 10,293 ms, TE 55 ms, FOV 22.4 cm, 2 × 2 × 2 mm^3^ voxels, 60 slices). From the collected images, FA maps were calculated using the commercially available diffusion tensor analysis package on the MR consol. Calculations were performed by a previously proposed method (Le Bihan et al. 2001).

### Preprocessing of T1-weighted structural data

Preprocessing of the T1-weighted structural data was performed using VBM2 software (Gaser 2007), an extension of SPM2 with default parameter settings (Gaser 2007). We used a scanner-specific customized GM anatomical template and prior probability maps from GM and WM images constructed from T1WI taken using the same scanner employed in our previous study (Takeuchi et al. 2010a). This step was considered because the contrast of T1WI obtained in the present study may have differed from the existing template, and because each scanner introduces specific non-uniformities in image intensity and inhomogeneities in the B0 field. T1WI of each subject was segmented into GM and WM partitions using the abovementioned customized GM and WM prior probability maps from our previous study. The resulting images included extracted GM and WM partitions in the native space. The GM partition was then normalized to the abovementioned customized GM probability map from the previous study. The normalization parameters determined from this initial step were then applied to the native T1WI. These normalized T1-weighted structural data were then segmented into GM and WM partitions (resulting in GM and WM partitions in the normalized space). In addition, we performed a volume change correction (modulation) by modulating each voxel with the Jacobian determinants derived from spatial normalization, allowing for the determination of regional differences in the absolute amount of GM/WM (Ashburner and Friston 2000). This procedure resulted in maps representing rGMV/rWMV. Subsequently, all images were smoothed by convolving them with an isotropic Gaussian Kernel of 10-mm full width at half maximum (FWHM). The resulting maps representing rGMV/rWMV were then used for the whole-brain analyses described below. We used the non-isotropic adjusted cluster-size test in the whole-brain analyses of rGMV/rWMV described below (Hayasaka et al. 2004). We used this test because it uses height and extent thresholds to determine significance, which is more sensitive compared to the condition when only the height threshold is used to determine significance (Poline et al. 1997). However, non-isotropic adjusted cluster-size tests should be applied to non-stationary data (i.e., not uniformly smooth), such as VBM data (Hayasaka et al. 2004). A higher smoothing value such as 12-mm FWHM is recommended for this non-isotropic adjusted cluster-size test to avoid anticonservativeness (Silver et al. 2010). Although the smoothing value of 12-mm FWHM was within the range of widely used smoothing values, it is relatively large. Thus, to increase the level of localization, we used a smoothing value of 10-mm FWHM in this study. However, the significance and insignificance of the results in this study were not affected by the use of this smoothing value. When 12-mm FWHM was applied, the corrected *P* values of this cluster-size test remained significant and changed from 0.020 to 0.028 in OFC in rGMV analysis (see “Effects of executive (dys)functions during everyday events on rGMV”), unchanged at 0.002 in the left temporal WM area in rWMV analysis (see “Effects of executive (dys)functions during everyday events on rWMV”), and changed from 0.003 to 0.002 in the right temporal WM area in rWMV analysis (see “Effects of executive (dys)functions during everyday events on rWMV”).

### Preprocessing of diffusion imaging data

Preprocessing and data analysis were performed using statistical parametric mapping software (SPM5; Wellcome Department of Cognitive Neurology, London, UK) and implemented in Matlab (Mathworks Inc., Natick, MA, USA).

Before normalization, the skull of the unsmoothed *b* = 0 images of all subjects was stripped by masking the images using a threshold of given signal intensity from the spatially smoothed (using 8 mm FWHM) *b* = 0 images of each participant. The threshold for skull stripping was the same for all subjects, and visual inspection confirmed that the skulls of the subjects were stripped without removing the brain parenchyma of the subjects. Smoothing for skull stripping was performed to prepare mask images for skull stripping the unsmoothed *b* = 0 images. The processed *b* = 0 images that proceeded to the next processing step were unsmoothed images.

Using affine and non-linear spatial normalization algorithms, the skull-stripped *b* = 0 images of each participant were normalized to a skull-stripped *b* = 0 image template created from data obtained using our scanner (see Takeuchi et al. 2010c for the creation of this template). Briefly, this original template was created from the images of the same 55 subjects whose data were also analyzed in this study. The original skull-stripped *b* = 0 image template was created as follows: (a) The skulls from the unsmoothed *b* = 0 images of the study subjects were stripped. (*b*) These skull-stripped unsmoothed *b* = 0 images were then spatially normalized to a skull-stripped T2 template in SPM5. (c) The normalized skull-stripped *b* = 0 image was then smoothed using a Gaussian kernel of 8 mm FWHM and finally averaged. By applying the parameters derived from normalization of the skull-unstripped *b* = 0 images of each participant, the FA map images of each participant were spatially normalized to create an image with 2 × 2 × 2 mm^3^ voxels. Finally, the normalized FA map images were spatially smoothed using a Gaussian kernel of 8 mm FWHM.

### Statistical analysis

Statistical analysis of the rGMV/rWMV data was performed using VBM5 software (Gaser 2007), which is an extension of SPM5. In this analysis, we only included voxels with a GM/WM value >0.05 to avoid possible partial volume effects. Here we examined the association between rGMV/rWMV and DEX scores as well as whether these associations differed between sexes. In whole-brain analysis, we used voxel-wise analysis of covariance (ANCOVA) in which sex difference was a group factor (using the full factorial option of SPM5). In this analysis, age, RAPM scores, total brain volume (total GM volume + total WM volume), and DEX scores were covariates. Age, RAPM scores, and DEX scores were modeled so that each covariate had a unique association with rGMV/rWMV for each sex (using the interactions option in SPM5), thus enabling investigation of the effects of interactions between sex and each covariate. On the other hand, total brain volume was not modeled in this manner, and a common effect of total brain volume on rGMV/rWMV was assumed for both sexes. The centering option was used to center these interactions. The main effects of DEX scores and the interaction between sex and DEX scores were assessed using t-contrasts.

Statistical analysis of FA data was performed using SPM5. In this analysis, we only included voxels with a FA value >0.2 because FA is more susceptible to errors emerging from partial volumes (Pfefferbaum and Sullivan 2003), and this FA cutoff value enabled us to separate WM from other tissues (Salat et al. 2005). We examined the association between FA and DEX scores and whether these associations differed between sexes. In whole-brain analysis, we used a voxel-wise ANCOVA in which sex difference was a group factor (using the full factorial option of SPM5). In this analysis, age, RAPM scores, number of b0 images, and DEX scores were covariates. Age, RAPM scores, and DEX scores were modeled so that each covariate had a unique association with FA for each sex (using the interactions option in SPM5), which enabled investigation of the effects of interactions between sex and each covariate. On the other hand, the number of b0 images was not modeled in this manner, and a common effect of total brain volume on FA was assumed for both sexes. The centering option was used to center these interactions. The main effects of DEX scores and the interaction between sex and DEX scores were assessed using t-contrasts.

### Statistical threshold in whole-brain analysis

In analysis of rGMV/rWMV, the level of statistical significance was set at *P* < 0.05, corrected, at the non-isotropic adjusted cluster level (Hayasaka et al. 2004) with an underlying voxel level at *P* < 0.0025. Non-isotropic adjusted cluster-size tests can and should be applied when cluster-size tests are applied to data known to be non-stationary (i.e., not uniformly smooth), such as voxel-based morphometry (VBM) data (Hayasaka et al. 2004). In this non-isotropic cluster-size test of random field theory, a relatively high cluster-determining threshold combined with high smoothing values of more than six voxels has been shown to lead to appropriate conservativeness in real data (Silver et al. 2010). In this test, under a high smoothing value, uncorrected threshold of *P* < 0.01 appears to lead to anti-conservativeness whereas uncorrected threshold of *P* < 0.001 appears to lead to slight conservativeness (Silver et al. 2010). We used a cluster-size test because of its sensitivity (Friston et al. 1996).

In FA analysis, regions with significance were inferred using cluster-level statistics (Friston et al. 1996). Only clusters with a *P* < 0.05 after correction for multiple comparisons at cluster size with a voxel-level cluster-determining uncorrected threshold of *P* < 0.001 were considered statistically significant.

### Statistical analysis of areas with a strong a priori hypothesis

For areas with a strong a priori hypothesis, the level of statistical significance was set at *P* < 0.05 with a small volume correction (SVC) for multiple comparisons (family-wise error) in the region of interest (ROI). The areas with a strong a priori hypothesis in this study were the bilateral OFC, which was hypothesized to play a key role in EFEEs and the genu of the corpus callosum, which connects the bilateral OFC and adjacent bilateral frontal regions (Huang et al. 2005). ROI for the bilateral OFC was constructed using the WFU PickAtlas Tool (http://www.fmri.wfubmc.edu/cms/software#PickAtlas) (Maldjian et al. 2003, 2004), and was based on the PickAtlas automated anatomical labeling atlas option (Tzourio-Mazoyer et al. 2002). The mask of the bilateral OFC was constructed by adding the mask images of regions of the orbital part of the superior frontal gyrus, the medial orbital part of the superior frontal gyrus, the orbital part of the middle frontal gyrus, and the orbital part of the inferior frontal gyrus using WFU PickAtlas Tool based on the Talairach Daemon option. The genu of the corpus callosum was constructed using the ICBM DTI-81 Atlas (http://www.loni.ucla.edu/).

## Results

### Behavioral data

Table 2 shows the average age, SD of age, and RAPM and DEX scores in males and females as well as statistical values (*P* values, *T* values, and effect sizes) for the comparison of these values between sexes. Males had significantly higher DEX scores than females. For complete data on the distribution of DEX scores in males and females see Table 3. The DEX and RAPM scores were not significantly correlated (simple regression analysis: *P* = 0.578, *t* = −0.557, *r* = 0.032). Although the average DEX score may appear small compared with the largest possible DEX score (80), we believe the relatively low score is characteristic of the questionnaire and does not indicate that there is no meaningful variances in DEX scores due to high EFEEs in this sample. This is because patients with Alzheimer’s disease have an average DEX score of 34.5 with a relatively high SD (Shinagawa et al. 2007). Furthermore, it should be noted that this is a subjective questionnaire and the scaling of the score may be different among subjects (Shinagawa et al. 2007). Furthermore, distribution of DEX scores in this study appeared to be normal, as can be seen in the Table 3, indicating that there are meaningful variances in this study (although, in the case of females, there may be a small reverse ceiling effect in several subjects).

**Table 2:** Demographic variables and statistical values for comparison between males and females

Measure	Males					Females					*P* value	*T* value	Effect size
	Mean	SD	Range	Kurtosis	Skewness	Mean	SD	Range	Kurtosis	Skewness			
Age	21.12	1.79	18–27	0.135	0.504	21.39	1.67	18–27	1.632	0.886	0.176	−1.36	−0.157
RAPM	28.37	3.37	18–36	−0.406	−0.229	27.93	3.81	18–36	−0.581	0.047	0.290	1.06	0.123
DEX	25.07	11.10	0–55	−0.356	0.262	18.93	9.94	0–50	−0.271	0.399	<0.001	5.01	0.580

**Table 3:** Distribution of DEX scores for males and females in our sample

	0–9	10–19	20–29	30–39	40–49	50–59
DEX male	11	46	53	39	15	3
DEX female	26	48	39	19	2	1

### Effects of executive (dys)functions during everyday events on rGMV

**Fig. 1:**
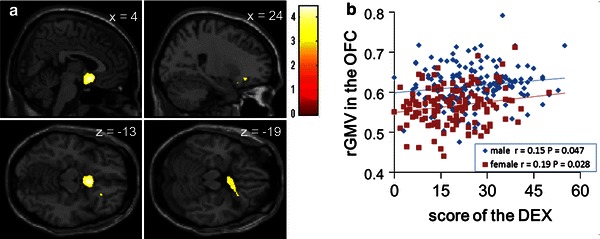
The associations between rGMV and EFEEs. **a** A region of positive association between rGMV and DEX scores (negative association between rGMV and EFEEs). Results are *P* < 0.05, corrected for multiple comparisons at the non-isotropic adjusted cluster level with an underlying voxel level of *P* < 0.0025, uncorrected. The region of significant correlation is overlaid on a “single-subject T1” SPM5 image. The regions with significant associations are seen mainly in the posterior and medial parts of OFC, which include BAs 25, 11, and 12. **b** The *panel* shows a scatter plot of the relationship between DEX scores and mean rGMV within a significant cluster in OFC. The *blue line* represents the regression line for males, and the *red line* represents the line for females. *r* values (correlation coefficients) and *P* values are for the correlation between DEX scores and mean rGMV within the significant cluster of OFC for males and females (simple regression analyses)

ANCOVA revealed an overall positive effect (regardless of sex) of DEX scores on rGMV in an anatomical cluster that mainly spread around the posterior medial part of OFC, which included Brodmann areas (BA) 25, 11, and 12 (MNI coordinates *x*, *y*, *z* = 4, 10, −13; *t* = 4.43; *P* = 0.020), corrected for multiple comparisons at the non-isotropic (non-stationary) adjusted cluster level with a cluster-determining uncorrected threshold of *P* < 0.0025 and raw cluster size of 4,164 mm^3^; Fig. 1a, b).

There was no overall negative effect of DEX scores on rGMV. In addition, ANCOVA revealed that there were no effects of the interaction between DEX scores and sex on rGMV. This was also true for analysis of areas with a strong a priori hypothesis using SVC. The significance (or insignificance) of these results was not affected when RAPM scores were removed from covariance. Note that lower DEX scores indicate higher functioning.

### Effects of executive (dys)functions during everyday events on rWMV

**Fig. 2:**
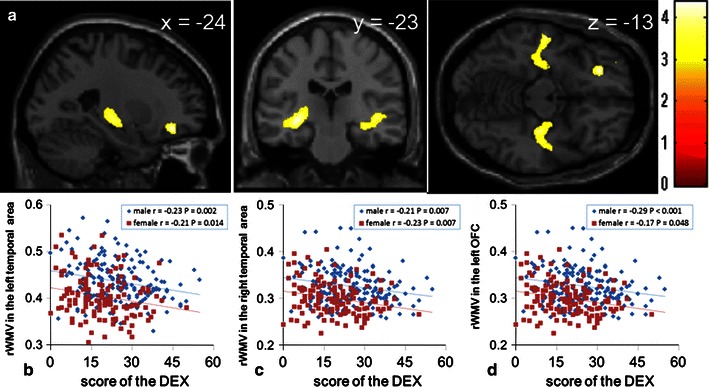
Associations between rWMV and EFEEs. Regions of negative association between rWMV and DEX scores (positive association between rGMV and EFEEs). Results are shown with a height threshold of uncorrected *P* < 0.0025 and an extent threshold of 500 voxels. Regions of significant correlation are overlaid on a “single-subject T1” SPM5 image. Regions with significant associations are shown in an area in the left OFC that is adjacent to BA 11 and in WM in the bilateral temporal areas. The association of OFC may look small under the threshold in the figure, but when the uncorrected threshold of *P* < 0.05 was used, the cluster including this area had an extent of 6,068 mm^3^. The *panels* show scatterplots of the relationships between DEX scores and mean rWMV **b** within the significant cluster of WM in the left temporal area, **c** within the significant cluster of WM in the right temporal area, and **d** within the cluster formed around the significant voxels (under the SVC) of OFC under the threshold of *P* < 0.0025, uncorrected. The *blue lines* represent the regression lines for males, and the *red line* represents the line for females. *r* values (correlation coefficients) and *P* values are for correlations between DEX scores and mean rWMV within the clusters (simple regression analyses)

ANCOVA analysis revealed an overall negative main effect (regardless of sex) of DEX scores on rWMV in the WM areas in the left hemisphere [i.e., close to the superior temporal sulcus (the superior temporal gyrus and the middle temporal gyrus); right hippocampus; and right parahippocampal gyrus] or WM structures mainly consisting of the inferior longitudinal fasciculus (ILF) and inferior fronto-occipital fasciculus (IFOF) (MNI coordinates *x*, *y*, *z* = −30, −23, −6; *t* = 4.39; *P* = 0.002 at the cluster level, raw cluster size 6,016 mm^3^; Fig 2a, b), and in the WM areas in the right hemisphere, that is close to the superior temporal sulcus (the superior temporal gyrus and the middle temporal gyrus), the right hippocampus and the right parahippocampal gyrus or the WM structures mainly consisting of ILF and IFOF (MNI coordinates *x*, *y*, *z* = 32, −20, −13; *t* = 3.97; *P* = 0.003, at the cluster level, raw cluster size 3,940 mm^3^; Fig 2a, c).

Among areas with a strong a priori hypothesis, SVC was employed and an overall negative effect (regardless of sex) of DEX scores on rWMV in the WM areas in the left OFC, which was adjacent to BA 11, was observed (SVC for areas with a strong a priori hypothesis: *x*, *y*, *z* = −24, 36, −13; *P* = 0.014 corrected for FWE at the voxel level within OFC, raw cluster size 600 mm^3^ under the threshold of *P* = 0.0025; Fig. 2a, d).

There was no overall positive effect of DEX scores on rWMV. ANCOVA revealed that there were no effects of the interaction between DEX scores and sex on rWMV. The significance (or insignificance) of these results was not affected when the RAPM scores were removed from covariance. Note that lower DEX scores indicate higher functioning.

When the males and females were analyzed separately using multiple regression analyses with the same covariates, among the abovementioned significant correlations, only the significant correlations observed in the left temporal WM area and the WM area of the left OFC remained significant when only the data from male were analyzed. However, as can be seen in Fig. 2b and d, similar associations between the DEX score and rWMV were also observed within these areas in females.

### Effects of executive (dys)functions during everyday events on FA

ANCOVA revealed that there were no significant overall effects (regardless of sex) of DEX scores on FA. In addition, ANCOVA revealed that there were no effects of the interaction between DEX scores and sex on FA. This was also true for analysis of areas with a strong a priori hypothesis using SVC, indicating that the association between WM structural integrity and EFEEs was not clear in this study.

## Discussion

Rather consistent with our hypothesis, our findings showed that individual EFEEs measured by DEX (lower scores indicate higher functioning) in a non-clinical sample were negatively associated with rGMV in OFC (BAs 25, 11, and 12) and positively with rWMV in left OFC (adjacent to BA 11). Furthermore, individual EFEEs were positively associated with rWMV in bilateral WM areas [i.e., close to the superior temporal sulcus (the superior temporal gyrus and the middle temporal gyrus), the right hippocampus, and the right parahippocampal gyrus] or bilateral WM structures mainly consisting of ILF and IFOF, the latter of which connects OFC and posterior regions as described below. Our findings suggest that GM and WM structures involving OFC and other regions play a key role in maintaining better EFEEs, and thus, the successful maintenance of functions during everyday life among non-clinical subjects.

OFC is anatomically connected to various regions. It obtains sensory information from regions such as the gustatory cortex and temporal cortex, affective information from the amygdala, and motivational information from the hypothalamus (Wallis 2007). The basic role of this region is considered to be the integration of such information to determine potential reward outcomes and to make these available for effective decision making and reward-related learning (Wallis 2007). Another proposed function of OFC is that it plays a role in response inhibition, although this may be at least partly explained by reward-related processes (failure to learn to inhibit behaviors that are no longer rewarded) (Wallis 2007). OFC dysfunction may lead to disruption of these cognitive processes and cause associated problems in some everyday events, such as the inability to make correct decisions, failure to learn socially appropriate rules, and failure to inhibit socially inappropriate behaviors.

Association between OFC structures and EFEEs is congruent with the fact that lesions in OFC lead to executive dysfunctions, especially those affecting everyday events. One example of this is the case of Elliot, a famous patient with an OFC lesion resulting from surgery for removal of the tumor (Damasio 1994; Eslinger and Damasio 1985). His lesions included BAs 25, 11, and 12 (Eslinger and Damasio 1985). He was still able to speak intelligently after damage to the bilateral OFC. Tests examining his intelligence and several other cognitive functions revealed average to superior ability. Even tests designed specifically to test frontal lobe processes related to executive function, such as working memory and rule-switching tasks, failed to reveal any deficits. Yet in real life situations he had serious difficulty making correct decisions. Within months of surgery he lost his job, wealth, divorced his wife, and lost contact with family and friends. In other cases involving damage to OFC, such as the cases of a countless number of lobotomy patients, executive dysfunctions such as disinhibition, irritability, and lability were observed (Damasio 1994).

The peak and main extent of the GM correlates of EFEEs were located in BA 25 or the subgenual cingulate gyrus. These areas had anatomical connections with a wide range of subcortical areas and are suggested to be involved in the regulation of these areas as well as in the utilization of signals from these regions in social norms and decision making; thus, dysfunction of this area may cause a wide range of problems in our everyday lives. A reduction in rGMV in BA 25 is seen in affective disorders (Drevets et al. 2008; Hajek et al. 2008) and schizophrenia (Fornito et al. 2009). Functional neuroimaging studies of post-traumatic stress disorder also revealed abnormal activity in this area related to exposure to traumatic remainders (Van Boven et al. 2009), and greater cortical thickness in this area is a stable marker of the recovery potential in this disease (Dickie et al. 2012). The tissue near this area (subgenual cingulate gyrus) shows increased hemodynamic activity during various emotional–behavioral tasks, including tasks involving sadness induction (George et al. 1995); exposure to traumatic reminders (Rauch and Drevets 2008); selecting sad or happy targets in an emotional go-no-go study (Elliott et al. 2000b); monitoring of internal states in individuals with attachment avoidant personality styles (Gillath et al. 2005); and extinction learning to previously fear-conditioned stimuli (Phelps et al. 2004). These findings suggest the role of this area in the automatic regulation of emotional behaviors (Drevets et al. 2008). Furthermore, this area is activated in response to a wide range of motivation- and reward-related cognitions, including response to the unpredictability of a reward (Berns et al. 2001), winning streaks (Elliott et al. 2000a), warm pleasant feelings (Rolls et al. 2008), pleasantness felt when eating chocolate (Small et al. 2001) and seeing the face of the person we love (Xu et al. 2011) or our own babies (Bartels and Zeki 2004), affiliative reward mechanisms (Young and Wang 2004), and charitable donations (Moll et al. 2006). This area is also activated in response to high risk (Van Leijenhorst et al. 2010). Damage to the subgenual sector of the anterior cingulate in monkeys impaired their ability to inhibit responses to a previously rewarded stimulus, and thus hindered reversal learning of a stimulus–reward association (Dias et al. 1996, 1997). In humans, it has been shown that impairments of reversal learning of previously rewarded responses are associated with damage involving the subgenual sector of the anterior cingulate (Owen et al. 1991; Rolls et al. 1994). The subgenual sector of the anterior cingulate appears critical for motor impulsiveness to affective stimuli, which reflects an inability to inhibit a pre-potent response to an affective stimulus such as a reward (Bechara et al. 2002). The activity of this area is also related to sleep (Rolls et al. 2003). The subgenual cingulate gyrus shares extensive anatomical connections with the amygdala, subiculum, hypothalamus, accumbens, ventral tegmental area (VTA), substantia nigra, raphe, locus ceruleus, periaqueductal gray and brainstem autonomic nuclei, and other areas of the orbitomedial PFC. Based on these previous studies as a whole, it is suggested that this region modulates both the behavioral response elicited through the ventral striatum (reward) and the emotional response triggered via the amygdala and other autonomic structures (Bechara et al. 2002) through these anatomical connections. It may also be associated with a function linking the signals from reward- and autonomic-related signals to social cognitions (Moll et al. 2006). Finally, this region is suggested to be involved in decision making by the representation of reward-related signals and the utilization of other autonomic signals (Ridderinkhof et al. 2004). As a whole this region may function as a link between higher-order cognitive norms and responses from subcortical areas to produce complex cognition in our everyday lives. Thus, defects in these mechanisms may cause a wide range of problems in our everyday lives as well as changes in emotional, behavioral, personality, motivational, and cognitive norms.

The negative association between rGMV in OFC and better EFEEs may be underlined by the fact that less rGMV is associated with increased and superior cortical development in the medial PFC (mPFC). As discussed in our previous study (Takeuchi et al. 2010b), increasing evidence has shown that with regard to GM structures in young adults, we cannot make the simple assumption that “the more, the better.” For example, normal cortical development after adolescence is characterized by cortical thinning, and the frontal lobe thins the most during late adolescence and early adulthood (Sowell et al. 1999). In other words, the more biologically developed one is the thinner the cortices are. Thus, thinner cortices in these areas may reflect matured cognitive functions. Furthermore, developmental studies of intelligence have shown that among same-age groups, children with the highest level of intelligence show the most vigorous cortical thinning in the mPFC during adolescence (Shaw et al. 2006). Another study of older children showed that thin cortices were associated with a more mature pattern of functional activation (Lu et al. 2009). Furthermore, longitudinal training and intervention studies in normal subjects have revealed decreased GMV in areas associated with cognitive functions involved in training a while after training was stopped (Boyke et al. 2008; Driemeyer et al. 2008) or after intensive training given over a short period (Takeuchi et al. 2011b, c). These phenomena may lead to positive associations between superior cognitive abilities and lesser rGMV in relevant regions in early adulthood. Consistent with this, cognitive functions in social and emotional realms are associated with lesser GM in the frontopolar cortex among young adults (Takeuchi et al. 2010b). While mPFC or the contingent ventromedial PFC plays an important role in empathy together with other regions (Rankin et al. 2006; Schulte-Rüther et al. 2011; Shamay-Tsoory et al. 2003, 2009), decreased rGM in mPFC, contingent ventromedial PFC, and precuneus is associated with increased empathy (Takeuchi et al. submitted). Furthermore, in certain other cases, thinned cortices have been associated with greater or increased cognitive functions (for a review, see Kanai and Rees 2011). Finally, amusia subjects have a thicker cortex in the right auditory cortex relative to musically intact controls (Hyde et al. 2007). This type of association may underlie the observed negative association between rGMV and better EFEEs. However, increased rWMV may indicate increased myelination in the associated regions, which in turn facilitates neural transmission in the area and neural transmission among the networks, leading to enhanced EFEEs. Alternatively, there is a possibility that the diminished OFC volume might lead to mutually disinhibitory cortical networks that then lead to higher cognitive functioning, as discussed previously (Jung et al. 2010). However, considering the critical importance of inhibitory cognitive functions on executive functions (Shallice 1988) and the clinical evidence of the positive contribution of OFC to better EFEEs, we believe that in the present study population, the reduced rGMV of the areas described is associated with better functioning in these areas as a result of cortical thinning.

The reason for the negative association between rGMV and EFEEs as well as between cognitive functions in the social and emotional realms in mPFC may have something to do with autistic traits and absence of synaptic pruning. It may be interesting to note that individuals with autism who have apparent compromised EFEEs and other related cognitive functions as well as high ability in certain areas, also have cortices with greater rGMV (for a review, see Brambilla et al. 2003). The cortices with greater GMV seen in individuals with autism are suggested to be because of absence of synaptic pruning (Hill and Frith 2003). Furthermore, even among normal samples, there are individual differences in traits associated with the autistic spectrum (Baron-Cohen et al. 2001). These individual trait differences may be because of absence of some degree of synaptic pruning. This concept may be consistent with the idea that autism and Asperger syndrome lie on a continuum, with Asperger syndrome seen as the ‘bridge’ between autism and normality (Baron-Cohen 1995). The reason for the positive association between superior cognitive functions and rGMV even in young adults may be explained in a complex manner by (a) whether superior cognitive functions are associated with autistic traits and (*b*) whether the region shows a developmental rGMV decrease with age (for example, the region around the superior temporal gyrus does not).

Discrepancies between our VBM findings and previous clinical VBM studies that have reported a negative association between executive functions and lower amounts of rGMV could be because of the different physiological bases that determine rGMV in clinical and non-clinical samples of young adults. Our VBM findings were not congruent with clinical VBM studies that have reported a negative association between specific types of executive functions in patients and GM in OFC (Takeuchi et al. 2010b). For example, a study showed that GM positively correlates with foresight in patients with schizophrenia; foresight is a type of executive function that occurs during everyday events (Eack et al. 2008). As discussed in that study (see Takeuchi et al. 2010b, for detail), the incongruency between clinical VBM studies and the study described above may be due to the following mechanisms: (1) among certain clinical samples, neuronal degeneration, including neuron loss (Baron et al. 2001; Thieben et al. 2002), leads to executive dysfunctions and reduced rGMV signal intensity in segmented GM images and (2) however, among non-clinical young adults, adaptive development underlined by increased synaptic pruning leads to better executive functions and reduced rGMV signal intensity in segmented GM images.

WM in the bilateral temporal areas, where rWMV is positively associated with EFEEs, involve a WM bundle connecting OFC and a WM bundle connecting the regions that play essential roles in reading information during social interactions, which in turn play important roles in EFEEs. EFEEs were positively associated with rWMV in WM areas [i.e., the superior temporal sulcus (the superior temporal gyrus and the middle temporal gyrus), right hippocampus, and right parahippocampal gyrus] or WM structures mainly consisting of ILF and IFOF. IFOF directly connects the frontal lobe (mainly OFC and other prefrontal regions) with the posterolateral temporal and occipital lobes (Catani et al. 2003; Fernández-Miranda et al. 2008; Martino et al. 2010). This tract connects many regions and may have various functions (Martino et al. 2010). EFEEs are considered to be part of these because frontal cognitive symptoms, such as dysexecutive problems, apathy, and personality changes are associated with degeneration of this tract (Kvickström et al. 2011). On the other hand, ILF consists of fiber connections between the occipital and anterior temporal cortex (Catani et al. 2003). ILF originates in the extrastriate areas of the occipital lobe and regions around the fusiform gyrus; it terminates in the lateral and medial temporal cortex in the region of the amygdala and parahippocampal gyrus (Catani et al. 2003). These regions and ILF play essential roles in intrapersonal communication (Takeuchi et al. 2011e) through the perception of face (fusiform gyrus) (for a review, see Kanwisher and Yovel 2006), a ‘brake’ in social situations (amygdala) (Amaral 2002), and processing of paralinguistic information in social contexts (parahippocampal gyrus) (Rankin et al. 2009). Finally, both bilateral superior temporal sulci are suggested to play a key role in social perception and speech or linguistic perception, which are essential for social communication (Milligan et al. 2007). As the reading of social cues and implicit information in social interactions is an essential part of EFEEs (Table 1), larger WMV in this area in subjects with higher EFEEs may aid efficient neural transmission and functioning.

Executive dysfunctions emerge from lesions in areas other than OFC in the frontal lobe, such as the dorsolateral PFC (DLPFC) and the anterior cingulate cortex (Mashour et al. 2005). Furthermore, items in DEX indicate a broad range of frontal functions (planning and strategy formation, inhibition, and control). As described by Stuss and Benson (1984, 1986), DEX samples broad areas of likely changes associated with dysexecutive syndrome and these changes include cognitive changes as well as emotional, motivational, and behavioral changes (Burgess et al. 1998). The broad range of functions may be subserved by different parts of the frontal lobe. Nevertheless, in our study, only OFC-related structures were identified as anatomical correlates of EFEEs measured by DEX. One possible cause of this (other than that the OFC structures are only anatomical correlates of EFEEs) was described when we mentioned the case of Elliot, the famous patient with OFC lesions including those in BAs 25, 11, and 12 (Damasio 1994; Eslinger and Damasio 1985). EFEEs are likely to be best supported by the functions of OFC, although a lack of statistical power may lead to negative findings in other frontal regions. In particular, certain executive functions are supposed to play a key role in working memory systems, capacity of which can be measured under laboratory test settings. DLPFC plays a key role in the executive functions of the working memory system (Baddeley 2003). However, DLPFC may play a relatively minor role in EFEEs, which partly correspond to the hot executive functions when compared to OFC, as described in “Introduction”. Consistent with this notion, in this study, moderate but significant negative correlations between higher DEX scores (lower EFEEs) and higher Stroop task scores (for details of procedures of this test, see Takeuchi et al. 2011c, 2012) were observed among the 301 subjects for whom all the relevant data could be obtained (*P* = 0.007, *r* = −0.154, simple regression analysis). Furthermore, there was a tendency toward a positive correlation between higher EFEEs, as measured by DEX, and larger rGMV in DLPFC as well as larger rWMV in the bilateral inferior frontal gyri (all *P* < 0.0025, uncorrected and >200 voxels) in this study.

Apart from the aforementioned limitation, this study has other limitations. First, similar to our previous studies (Takeuchi et al. 2010a, c), we used young healthy subjects with an advanced educational background. Limited sampling of the complete range of intellectual abilities is a common hazard when sampling from college cohorts. Whether our findings would also hold across the full range of population samples and a normal distribution must be determined with larger and more representative samples. The second limitation is the manner in which DEX was used. A previous study (Chan 2001a) supported the validity of DEX in a normal population and our data showed that DEX scores followed a normal distribution pattern in our sample, suggesting that executive (dys)functions during everyday life events are normally distributed among non-clinical samples. However, DEX has mainly been used in clinical populations and its validity, when administered to normal populations, may be weak. Furthermore, DEX has two versions, self- and informant-reported questionnaires. In other studies using DEX, sometimes only the self-reported version (Amieva et al. 2003; Bodenburg and Dopslaff 2008; Heffernan et al. 2004) and sometimes only the informant-reported version (Chaytor and Schmitter-Edgecombe 2007; Krabbendam et al. 1999) was used, yet in other cases both versions were used (Burgess et al. 1998; Odhuba et al. 2005). Some studies have reported that the informant-reported version correlated better with psychological measures, such as other executive functions, general cognitive functions, and perception, than the self-reported version (Burgess et al. 1998; Evans et al. 1997). On the other hand, other studies have reported that the self-reported version correlated better with psychological measures, such as executive function and general cognitive functions (Odhuba et al. 2005; Wood and Liossi 2006). However, who reports symptoms may not be as important for non-clinical samples as for samples of patients with severe brain injuries. This is because patients with severe brain injuries may lack insight into their own symptoms (Prigatano 1991). Consistent with this idea, in clinical samples, informants give higher DEX scores (indicating severe dysexecutive symptoms) than subjects, whereas in non-clinical samples subjects give higher DEX scores than informants (Burgess et al. 1998), suggesting that only among clinical samples do subjects underestimate the severity of their own dysexecutive problems. Since one study (Burgess et al. 1998) reported a difference in the scores for the self- and informant-reported versions of DEX, administration of both the versions of DEX might have added to this study. Finally, another limitation is the automated preprocessing procedure. While automated preprocessing in VBM procedures facilitates the processing of large amounts of data at the whole-brain level without limiting the choice of regions to a priori hypothesized selections, it may suffer from more preprocessing errors compared with manual volumetry (Kennedy et al. 2009), which is the trade-off we selected. We nevertheless included more than 300 subjects, and problems randomly caused by automated processing are less of a concern as they do not correlate with EFEEs (although analysis may lose its sensitivity). Furthermore, while OFC was the ROI in this study, it is an extensive area, and its different areas are known to have different functions (e.g., Kringelbach 2005). Thus, the use of ROI analysis that use total or mean values of the entire ROI (instead of voxel-based ROI analysis regardless of whether performed manually or by an automated process) may be slightly problematic for the purpose of this study because averaging the signals in functionally inhomogeneous areas may erase the effect of interest (Poldrack 2007), whereas a functional ROI cannot be used in this study.

This is the first study to investigate the association between brain structures and EFEEs measured by a behavioral questionnaire administered to a non-clinical sample. Previous neuropsychological studies have suggested that lesions in the OFC can lead to selective impairment of EFEEs, and cause serious problems in everyday life. Our study showed that, even among non-clinical subjects, structural variations related to the OFC together with those of other regions that underlie individual EFEEs.
